# Family mapping of previously identified patients with pathogenic or likely pathogenic *ALPL* variants using predictive genotyping and detailed phenotyping approach: the FAME case-control study

**DOI:** 10.1093/jbmrpl/ziaf034

**Published:** 2025-02-27

**Authors:** Tatiane Vilaca, Fatma Gossiel, Sophie Delaney, Duncan Baker, Sylvia Keigwin, Richard Eastell, Meena Balasubramanian

**Affiliations:** Division of Clinical Medicine, University of Sheffield, Sheffield S10 2RX, United Kingdom; Division of Clinical Medicine, University of Sheffield, Sheffield S10 2RX, United Kingdom; Sheffield Diagnostic Genetics Service, North East and Yorkshire Genomic Laboratory Hub, Sheffield Children’s NHS Foundation Trust, Sheffield S10 2TH, United Kingdom; Sheffield Diagnostic Genetics Service, North East and Yorkshire Genomic Laboratory Hub, Sheffield Children’s NHS Foundation Trust, Sheffield S10 2TH, United Kingdom; Sheffield Diagnostic Genetics Service, North East and Yorkshire Genomic Laboratory Hub, Sheffield Children’s NHS Foundation Trust, Sheffield S10 2TH, United Kingdom; Division of Clinical Medicine, University of Sheffield, Sheffield S10 2RX, United Kingdom; Division of Clinical Medicine, University of Sheffield, Sheffield S10 2RX, United Kingdom; Sheffield Children’s NHS Foundation Trust, Sheffield S10 2TH, United Kingdom

**Keywords:** hypophosphatasia, alkaline phosphatase, ALPL gene, family mapping, brief pain inventory, modified hypophosphatasia impact survey, pyridoxal 5-phosphate (PLP), phosphate, musculoskeletal pain, fractures

## Abstract

Hypophosphatasia (HPP) is an inborn error of metabolism caused by loss-of-function variants in the *ALPL* gene, which encodes the tissue nonspecific isozyme of alkaline phosphatase (ALP). There is no typical phenotype in adults. We used a genotyping first approach to determine whether *ALPL* pathogenic variants were associated with musculoskeletal symptoms, mineral metabolism abnormalities, and an impact on quality of life. We recruited individuals with a pathogenic (or likely pathogenic) variant in *ALPL* gene (*n* = 26) and their relatives (*n* = 44). We performed genetic tests and compared the relatives with positive (*n* = 20) and negative (*n* = 24) genetic test. We applied standard questionnaires and physical tests (Brief Pain Inventory [BPI]; Western Ontario and McMaster Universities Arthritis [WOMAC]; Modified Hypophosphatasia Impact Patient Survey; Short Form of 36 Survey [SF-36]; and the Short Physical Performance Battery). In fasting blood samples, we measured creatinine, calcium, phosphate (P), parathyroid hormone (PTH), ALP, bone ALP, 25OHD-, 1,25(OH)2D, CTX, type 1 procollagen N-terminal peptide (PINP), osteocalcin, and tartrate-resistant acid phosphatase5b (TRACP5b). Relatives with positive genetic test had lower ALP (IU/L) [32.5(12.8) vs 87.8(32.6) *p* < .001], bone ALP (ng/mL) [6.3(4.3, 9.8) vs 17.5 (13.12-25.7) *p* < .001], PTH (pg/L) [28.6(20.6, 38.1) vs 40.05(25.7, 52.3) *p* = .03], and higher PLP(nmol/L) [162.0 (91.75, 337.5) vs 37.5 (18.25, 60.5) *p* < .001] and P(mmol/L) [1.36 (0.18) vs 1.05 (0.2) *p* < .001]. We did not find significant differences in fractures or musculoskeletal features between the groups. Greater pain scores were observed on BPI in relatives with positive genetic tests, and bone and muscle pain were more often reported by this group, but statistical tests were not significant. No differences were found in physical performance or quality of life. In conclusion, we assessed relatives of individuals with pathogenic or likely pathogenic variants in the *ALPL* gene regardless of the presence of signs and symptoms. Biochemical abnormalities were more common in gene-positive relatives, but the prevalence of musculoskeletal symptoms was comparable in relatives with positive and negative genetic tests.

## Graphical Abstract

**Graphical Abstract:**
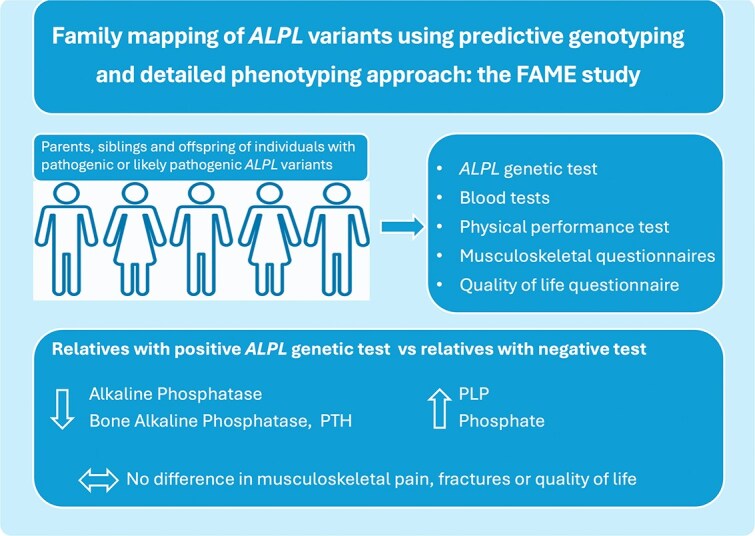

## Introduction

Hypophosphatasia (HPP) is the inborn error of metabolism caused by pathogenic or likely pathogenic variants in the *ALPL* gene.^1^^,^^2^ The gene encodes the tissue nonspecific isoenzyme of alkaline phosphatase (TNSALP), and pathogenic variants reduce enzyme activity. The result is defective mineralization of bone and/or teeth.^3^ The disease is characterized by extreme clinical heterogeneity and is historically classified into 6 clinical phenotypes based on age at of onset: perinatal HPP; perinatal benign HPP; infantile HPP; childhood or juvenile HPP; adult HPP; and odontohypophosphatasia without any obvious skeletal manifestations.^1^^,^^4^^,^^5^

HPP in adults varies clinically from mild to severe. Low serum ALP activity and elevated serum pyridoxal 5′-phosphate (PLP) levels suggest the diagnosis, which may be confirmed by clinical features and/or identifying pathogenic variants in *ALPL* by genetic testing.^6^^,^^7^ However, vitamin B6 deficiency may prevent the increase in PLP levels. Metatarsal and femoral fractures are often the initial manifestations in adults, and the condition may be misdiagnosed as osteoporosis, even though bone mineral density is frequently normal or even increased.^8^^,^^9^ The most widely used treatment for osteoporosis is bisphosphonates. These drugs are pyrophosphate analogs, and they may further inhibit bone mineralization in HPP, potentially making patients with HPP more susceptible to stress fractures.^10^ Furthermore, prolonged bisphosphonate treatment for osteoporosis is associated with an increased risk of atypical femoral fractures.^10^^,^^11^ Previous case reports suggested that bisphosphonates use in adults with HPP can increase such fractures, and *ALPL* variants have been identified in patients with atypical femoral fractures.^10^^,^^11^ The early diagnosis might prevent someone from receiving bisphosphonates and being exposed to a higher risk of atypical femoral fractures. Furthermore, identifying a possible underlying cause for symptoms like chondrocalcinosis, enthesopathy, and musculoskeletal pain might avoid additional investigation and promote an appropriate approach, such as pain management. Another important outcome is a better-informed recurrence risk discussion despite the variability in phenotype and poor genotype/phenotype correlation for reproductive decision-making in those with the familial variant.

This study aimed to examine the relationship between the presence of a pathogenic or likely pathogenic variant in *ALPL* and musculoskeletal symptoms, laboratory tests, and other aspects of general health status in relatives of individuals with a pathogenic or likely pathogenic variant in *ALPL* using family mapping and a predictive genotyping approach. In this approach, we performed the genetic test, first looking for the family variant in all the relatives, irrespective of the presence of biochemical abnormalities or clinical signs and symptoms. Relatives with pathogenic or likely pathogenic variants in *ALPL* were classified as the cases, while relatives without the variant were considered as controls.

## Materials and Methods

This is a single-centre, cross-sectional, observational, case-control study conducted at Sheffield Children’s Hospital and Sheffield Teaching Hospital in Sheffield, UK.

Patients with pathogenic or likely pathogenic *ALPL* variants were invited to participate as probands.^12^^,^^13^ Variants were classified according to ACGS Best Practice Guidelines for Variant Classification in Rare Disease 2024.^12^^,^^13^ We invited patients who had previously taken part in research studies (including RUDY, Rare Undiagnosed Disease StudY)^14^^,^^15^ or were in clinical follow-up. Participants were recruited between March 2022 and July 2023.

Probands were asked to pass on a standardized letter to their first- and second-degree relatives requesting their permission to be contacted by the research team regarding possible participation in the FAME study (*FA*mily *M*apping of pr*E*viously identified HPP patients using predictive genotyping and detailed phenotyping approach). We investigated parents, siblings, and adult offspring (≥16 yr) at first or second degree. We excluded potential participants with variants of unknown significance and individuals younger than 16 yr old.

The study was approved by the South Central—Oxford A Research Ethics Committee (REC 21/SC/0376). All participants provided written informed consent. All participants (probands and relatives) underwent the whole assessment except for the genetic test performed only on the relatives, as the probands already had a genetic test confirming the presence of the pathogenic or likely pathogenic *ALPL* variant. A 3-generation family pedigree was obtained from each participant. We collected fasting blood samples and measured creatinine, calcium, albumin, PTH, total and bone ALP, and 25-hydroxyvitamin D with autoanalyzer (Cobas c702, e411 and e602, Roche Diagnostics). We used the IDS iSYS multidiscipline automated chemiluminescence immunoassay (Immunodiagnostic Systems) to measure CTX and N-terminal propeptide of type I collagen (PINP), osteocalcin, tartrate-resistant acid phosphatase (TRACP5b), IGF1, and 1,25(OH)_2_D. We used a Chromsystems kit to derivatise and measure the PLP by HPLC with fluorescence detection. All interassay CVs were <6%. We used targeted Sanger sequencing to investigate the familial variant.

We used the Short Physical Performance Battery (SPPB), a validated, widely used test that assesses strength in lower limbs, gait, and balance to evaluate physical performance. SPPB includes 3 tests: the repeated chair standing test, the balance test, and the 8-foot walk course. These 3 objective measures are scored from 0 to 4, with higher scores indicating greater physical function.

We used standardized and validated questionnaires to characterize musculoskeletal symptoms.^16–18^ All participants completed the following 5 questionnaires: a medical history and lifestyle questionnaire to assess demographics and clinical risk factors for fractures, Western Ontario and McMaster Universities Arthritis Index (WOMAC) questionnaire,^17^ Brief Pain Inventory (BPI) questionnaire,^16^ a Short Form of 36 SF-Survey (SF-36),^18^ and the modified Hypophosphatasia Impact Patient Survey (HIPS), a questionnaire to assess the symptoms of HPP including fractures, tooth loss, and spine and joint problems.^19^

This is an exploratory study based on family mapping. Therefore, power calculations were not performed. The results from the various assessments were compared between cases (relatives gene variant positive) and controls (relatives gene variant negative).

Analyses were performed using IBM SPSS Statistics (version 29.0, IBM Corp.) and GraphPad Prism (version 10.2.3 GraphPad Software Inc) for Mac. All variables included in the study underwent testing for normal distribution (Kolmogorov-Smirnov test). For variables that exhibited a normal distribution, parametric tests, specifically the Student *t*-test for 2 groups. Conversely, non-normally distributed variables were subjected to nonparametric tests, such as the Mann-Whitney *U* test for 2 groups. In these analyses, we used linear regression to adjust for age and sex. Because the age distribution of bone turnover markers is quadratic, we included an age-squared term. For categorical variables, we used the chi-square test as the primary method. Additionally, when sample sizes were limited, the Fisher exact test was employed as a more appropriate alternative to assess significance. A *p*-value less than .05 was considered significant. The results are reported as mean and SD [mean (SD)] for normally distributed variables and median and interquartile range for non-normally distributed variables [median (IQR)].

## Results

We recruited 70 participants: 26 probands (index cases with known pathogenic or likely pathogenic variants in *ALPL*) and 44 relatives, all 16 yr old or older. Eighteen probands had *ALPL* pathogenic variants, and 8 had likely pathogenic variants. Twenty relatives had a positive genetic test for the *ALPL* variant (10 pathogenic variants, 10 likely pathogenic variants), and 24 had a negative test. The list of variants (Sup table 1) and number of participants in each family (Sup Figure 1) and with each variant are shown in Supplementary Material  S Figure 2 and S Table 1. All the participants with positive tests were heterozygous. The 26 families presented 17 variants. Table 1 shows the characteristics of the FAME study participants, including probands. No difference was found between the groups.

**Table 1:** Characteristics of the FAME study participants (probands, relatives with positive test for *ALPL* variants and relatives with negative test to for *ALPL* variants).

	Probands (*n* = 26)	Positive relative (*n* = 20)	Negative relative (*n* = 24)
**Age (yr)**	44.3 (±14.7)	42.7 (±18.2)	49.3 (±21.4)
**Height (m)**	1.65 [1.60, 1.72]	1.61 [1.56, 1.68]	1.69 [1.59, 1.75]
**Weight (kg)**	73.55 [64.2, 85.5]	81.25 [64.7, 102.2]	82.5 [65.7, 104.9]
**Sex (female/total), *N* (%)**	16 (62)	14 (70)	14 (58)
**Ethnic background**			
**White, *N* (%)**	26 (100)	17 (70)	24 (100)
**Mixed**	0	3	0
**Alcohol > 14UI/w, *N* (%)**	4 (15)	2 (10)	2 (8)
**Smoke, *N* (%)**	6 (23)	4 (20)	7 (29)
**Physical activity**			
**Very inactive, *N* (%)**	10 (38)	6 (30)	5 (20)
**Active everyday, *N* (%)**	11 (42)	11 (55)	14 (58)
**Regular formal exercise, *N* (%)**	3 (12)	2 (10)	4 (17)
**Daily strenuous exercise, *N* (%)**	2 (8)	0	0

Normally distributed variables are reported as means (±SD), and non-normally distributed as medians [interquartile range]. Categorical variables are reported as number of individuals and percentage *N* (%).

Table 2 shows the comparison of BPI, WOMAC, and SF-36 summary scores between relatives with positive and negative test to *ALPL* pathogenic or likely pathogenic variants. Pain scores on BPI here higher and bone and muscle pain were more often reported by relatives with positive genetic test, but statistical tests did not detect group differences (Table 2). Within the cohort of relatives with positive genetic tests (*n* = 20), 17 individuals, 85%, reported at least one symptom listed in the HIPS questionnaire. The most common was fractures, reported by 13 relatives (65%), followed by pain in several sites: limiting joint pain was reported by 12 (60%), bone pain by 10 individuals (50%), and muscle pain by 9 (45%). Seven individuals (35%) reported muscle weakness, 5 (25%) reported joint swelling, and 3 (15%) reported hypermobility. Tooth abnormalities were also highly prevalent. Although premature child tooth loss is a classical HPP dental abnormality, it was reported only by 5 individuals (25%), while adult tooth problems were more frequent. Excessive cavities were reported by 8 individuals (40%), loss of adult teeth by 7 (35%), and tooth abscesses by 6 (30%). The full comparison of the HIPS survey between individuals with positive and negative genetic tests is reported in Table S2. In Table 2, we show the comparison of fractures, hypermobility, pain, and dental abnormalities.

**Table 2:** Comparison of BPI, WOMAC, and SF-36 summary scores, fractures, pain, and dental abnormalities reported in HIPS between relatives with positive and negative *ALPL* pathogenic or likely pathogenic variants test.

	Positive	Negative	*p* value
**BPI (*n* = 42)**			
**Pain severity score**	3.75 [1.5, 5.75]	1.25 [0, 4.25]	.055
**Pain interference score**	4.2 [0.4, 8.0]	1 [0, 5.2]	.078
**WOMAC (*n* = 44)**			
**Total score**	39 [3, 65.75]	19.5 [3.75, 43.5]	.247
**SF-36 (*n* = 44)**			
**Physical health score**	38.9 (±15.5)	41.5 (±12.3)	.543
**Mental health score**	40.9 (±13.7)	44.6 (±12.8)	.364
**HIPS (*n* = 44)**			
**Have you had a fracture (yes), *n* (%)**	13 (65)	11 (45.8)	.2
**How many fractures?, *n* (%)**	1.15 (1.2)	0.75 (0.9)	.23
**Bone pain, *n* (%)**	10 (50)	6 (25)	.086
**Hypermobility, *n* (%)**	3 (15)	1 (4.2)	.237
**Joint swelling, *n* (%)**	5 (25)	3 (12.5)	.249
**Joint pain, *n* (%)**	10 (50)	10 (41.7)	.58
**Limiting joint pain, *n* (%)**	12 (60)	9 (37.5)	.137
**Muscle weakness, *n* (%)**	7 (35)	3 (12.5)	.147
**Muscle pain, *n* (%)**	9 (45)	5 (20.8)	.08
**Premature child tooth loss, *n* (%)**	5 (25)	3 (12.5)	.436
**Tooth abscess, *n* (%)**	6 (30)	3 (12.5)	.261
**Excessive cavities, *n* (%)**	8 (40)	4 (16.7)	.084
**Loss of adult teeth, *n* (%)**	7 (35)	7 (29.2)	.679

Normally distributed variables are reported as means (±SD), and non-normally distributed as medians [interquartile range]. Categorical variables are reported as number of individuals and percentage *N* (%).

SPPB showed no difference in performance between relatives with genetic test positive or negative when we compared the SPPB scores [total score 10.5 (9, 12) vs 11.5 (9.3, 12) *p* = .326] nor when we assessed each test individually.

**Figure 1:**
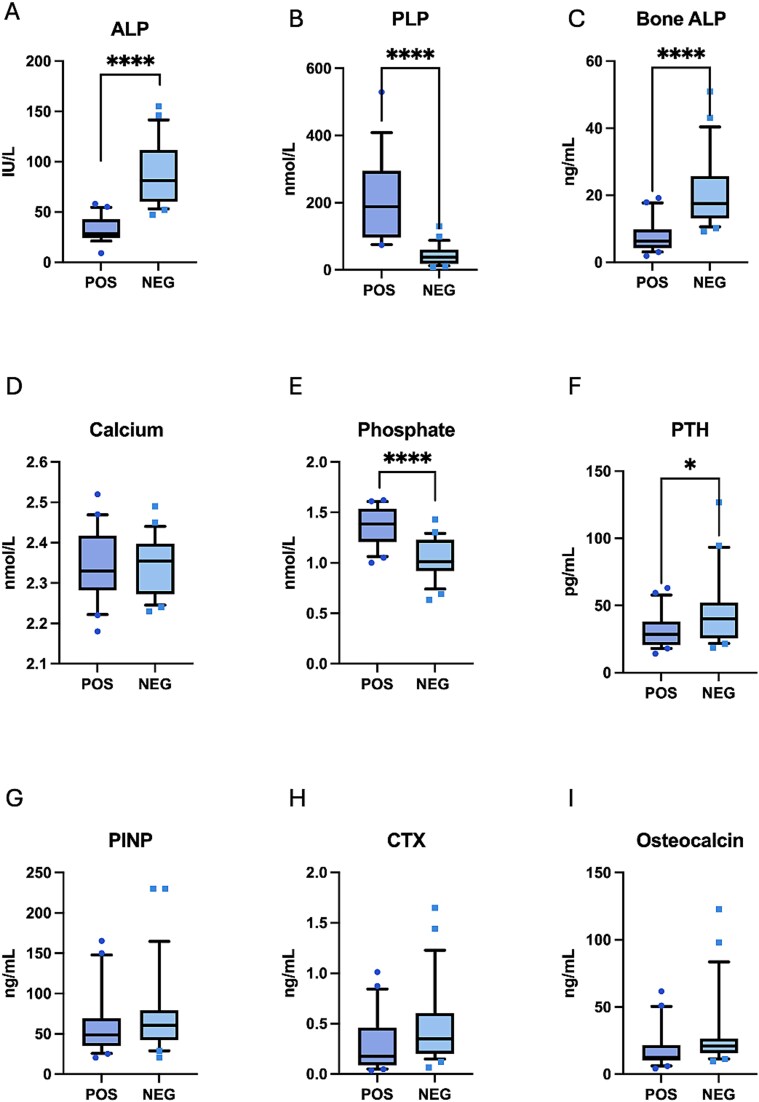
Comparison of mineral metabolism and bone turnover markers in relatives with positive (POS) and negative (NEG) genetic test for *ALPL* variant. ALP (alkaline phosphatase, A), PLP (pyridoxal5-phosphate, B), bone ALP (bone alkaline phosphatase, C), calcium (C, D), phosphate (D, E), PTH (E, F), PINP (type 1 procollagen N-terminal peptide, G), CTX (H), osteocalcin (I).^*^p < .05,^*^^*^^*^^*^p < .0001.

Table 3 shows the comparison of mineral metabolism and bone turnover markers between relatives with positive and negative test for pathogenic or likely pathogenic *ALPL* variants. Relatives with positive genetic test had higher phosphate and PLP and lower ALP, bone ALP, creatinine, and PTH (Table 3 and Figure 1). In unadjusted analysis, CTX and osteocalcin were lower in relatives with *ALPL* pathogenic or likely pathogenic variants. However, when we adjusted for age and sex, CTX and osteocalcin were no longer different between the groups. Calcium, 25OH vitamin D, 1,25 (OH)_2_ vitamin D, PINP, TRACP5b and IGF-1, and eGFR did not differ between the groups (Table 3 and Figure 1). Two relatives in the NEG group had eGFR < 60 mL/min.1.73 m^2^.

**Table 3:** Comparison of mineral metabolism and bone turnover markers between relatives with positive and negative test for pathogenic or likely pathogenic *ALPL* variants.

	Positive relative	Negative relative	*p*	Adjusted *p*
**ALP (IU/L)**	32.5 (±12.8)	87.8 (±32.6)	<.0001	
**PLP (nmol/L)**	204.5 [102.25, 325]	37.5 [18.25, 60.5]	<.0001	
**Calcium (nmol/L)**	2.34 (±0.09)	2.34 (±0.07)	.88	
**Phosphate (nmol/L)**	1.36 (±0.18)	1.04 (±0.20)	<.0001	
**PTH (pg/mL)**	28.6 [20.6, 38.1]	40.05 [25.7, 52.3]	.03	
**Creatinine (umol/L)**	70.4 (±10.6)	79.8 (±19.5)	.048	
**eGFR EPI (mL/min/1.73 m^2^)**	90 (±81.9)	88 (±66.5.9)	.22	
**IGF-1 (ng/mL)**	189.12 (±98.12)	155.4 (±72.4)	.20	
**25 OH D (ng/mL)**	20.4 (±8.2)	20.1 (±8.9)	.91	
**1,25OH_2_D (ng/mL)**	51.5 (±12.5)	52.3 (±17.0)	.86	
**PINP (ng/mL)**	48.8 [34.8, 69.3]	60.8 [42.3, 79.3]	.36	.38
**CTX (ng/mL)**	0.176 [0.0873, 0.460]	0.349 [0.202, 0.604]	.04	.12
**TRACP5b (U/L)**	2.79 [1.9, 3.4]	2.9 [2.3, 3.6]	.52	.8
**Bone ALP (ng/mL)**	6.3 [4.3, 9.8]	17.5 [13.1, 25.7]	<.0001	<.001
**OC (ng/mL)**	12.6 [10.2, 21.7]	20.9 [15.8, 26.6]	.018	.08

Normally distributed variables are reported as means (±SD), and non-normally distributed as medians [interquartile range]. Abbreviations: ALP, alkaline phosphatase; PLP, pyridoxal 5′-phosphate; PINP, type I procollagen N-terminal peptide; TRACP-5b, tartrate-resistant acid phosphatase.

## Discussion

Family mapping identified 20 individuals with an *ALPL* pathogenic or likely pathogenic variant. The mean age was 42.7, range 16-74. The clinical spectrum varied from asymptomatic to substantially affected by musculoskeletal symptoms, such as fractures, bone, and muscle pain. The increasing availability of genetic tests will likely increase the detection of individuals with *ALPL* pathogenic variants, especially those who have not presented suggestive phenotypes like metatarsal fractures. HPP diagnosis remains a challenge despite recent advancements in genomic testing due to poor genotype-phenotype correlation and the nonspecific nature of the clinical presentation.^7^

No previous studies have used the genotyping first approach and standardized questionnaires to characterize relatives of individuals with *ALPL* pathogenic or likely pathogenic variants. By genotyping first, we investigate the presence of the variant regardless of biochemical or clinical abnormalities. When we characterized the relatives of individuals with *ALPL* pathogenic or likely pathogenic variants, we investigated a cohort with similar genetic and environmental backgrounds. We used standardized questionnaires to compare the prevalence of musculoskeletal features in individuals with and without *ALPL* pathogenic or likely pathogenic variants. Previous surveys have reported that fractures and pain were the most prevalent features associated with *ALPL* variants. For example, Weber et al. showed that participants with HPP reported bone (82%), joint (73%), and muscle (53%) pain, and 86% reported fractures.^19^

Similarly, Seefried et al.^4^ showed that 62% of participants reported fractures or pseudofractures, 67% reported pain, and 28% reported muscular symptoms. However, fractures and musculoskeletal symptoms are not specific and are frequently found in the general population. We did not find significant differences in fractures or musculoskeletal features between the groups, but greater pain and pain interference scores were observed on BPI in relatives with positive genetic tests, and bone and muscle pain were also more often reported by this group in the HIPS survey. The consistency of the findings suggests that these features could be associated with *ALPL* variants, and a greater sample size could detect a difference. We found no difference in SF-36 general scores between the groups or in the physical performance test.

As expected, relatives with *ALPL* variants had lower ALP and greater PLP than relatives without the variant.^15^ Low ALP remains the main hallmark of diagnosis as it raises suspicions of abnormalities in *ALPL*. However, ALP reference intervals vary, which might influence the diagnosis. Notably, liver abnormalities may raise ALP levels, leading to normal ALP levels in people with pathogenic or likely pathogenic variants in *ALPL*. Moreover, some growth plates can still close up to 30 yr, increasing ALP levels up to this age. In a recent cohort, the ALP level of 43 IU/L was suggested as the threshold for detecting *ALPL* variants.^15^ Therefore, normal levels of ALP do not entirely exclude the presence of *ALPL* variants.

In addition, we also found lower PTH (but no difference in eGFR) and higher phosphate in individuals with positive genetic tests, as well as similar levels of calcium and vitamin D. The reason for the higher phosphate levels, often higher than the upper limit of the reference interval, is unclear. Low PTH levels might contribute. In children, a high renal tubular threshold maximum for phosphorus per glomerular filtration rate has been reported.^20^ Therefore, more research is needed to clarify mineral metabolism in adults with pathogenic or likely pathogenic variants in *ALPL*.

After adjustment for age and sex, we found no difference in CTX, TRAC5b, PINP, and osteocalcin between the groups. López-Delgado et al.^21^ reported lower CTX and PINP in adults with low ALP compared to healthy controls. In contrast, Desborough et al.^15^ reported higher TRACP5b and CTX in participants with positive genetic tests compared to postmenopausal women with low mineral density, most of them in treatment with bisphosphonates. However, these results are difficult to interpret because in the López-Delgado study, only 50% of the participants had the HPP diagnosis confirmed by genetic testing, and the authors suggest that low bone turnover is associated with low ALP levels.^21^ On the other hand, in the Desborough study, most of the participants in the control group were taking bisphosphonates. Because these drugs are known to reduce BTM, this might have affected the results, as most of the control group was expected to have lower bone turnover due to the use of antiresorptive drugs.^15^ In a Danish cohort, 40 participants with HPP on clinical follow-up were sex- and age-matched to healthy controls, PINP was 21% lower in the group with HPP, and there was no difference in osteocalcin and CTX. Similarly to our study, bone ALP was 3 times lower in participants with HPP.^22^ Therefore, the decrease in bone ALP is a consistent finding in individuals with pathogenic or likely pathogenic variants in the *ALPL* gene. In contrast, the results for other bone turnover markers are less consistent.

This study has limitations. Although the invitation to take part was open to any proband’s adult relative, the enrolment in the study was voluntary, and we only had information from the relatives who decided to take part. Therefore, the family mapping could be deemed relatively incomplete. Gene-negative relatives were not matched by age and gender to gene-positive relatives, but age and gender did not differ between the groups. We did not collect urine samples, and we did not investigate renal phosphate handling.

However, this study also has strengths. We comprehensively characterized features associated with HPP in families affected by pathogenic or likely pathogenic variants in *ALPL*. We used several biochemical and physical performance tests and validated standardized questionnaires to characterize the groups. We compared family members genetically and environmentally matched for other factors.

In summary, we assessed relatives of individuals with pathogenic or likely pathogenic variants in the *ALPL* gene regardless of the presence of signs and symptoms. Performing the genetic test on family members identified more individuals with biochemical abnormalities, but the prevalence of musculoskeletal symptoms was similar in relatives with positive and negative genetic tests. Physical function and quality of life were also comparable between these groups. While musculoskeletal features were nonspecific, biochemical abnormalities, such as lower levels of ALP and higher levels of PLP and phosphate, were associated with pathogenic or likely pathogenic *ALPL* variants. These results suggest that early *ALPL* genetic test, regardless of the presence of symptoms, did not increase the detection of symptomatic individuals.

## Supplementary Material

FAME_study-Supplementary_material_1_10Dec24_ziaf034

FAME_study-Supplementary_material_2_ziaf034

## Data Availability

The data from this study may be made available from the corresponding author upon reasonable request.
