# Supplementary material for: Family mapping of previously identified patients with pathogenic or likely pathogenic ALPL variants using predictive genotyping and detailed phenotyping approach: the FAME case-control study
Source: JBMR Plus. 2025 Feb 27;9(5):ziaf034. doi: 10.1093/jbmrpl/ziaf034 (PMC11993272; doi:10.1093/jbmrpl/ziaf034)
Supplement: FAME_study-Supplementary_material_1_10Dec24_ziaf034 [file fame_study-supplementary_material_1_10dec24_ziaf034.docx]

FAME study – supplementary material


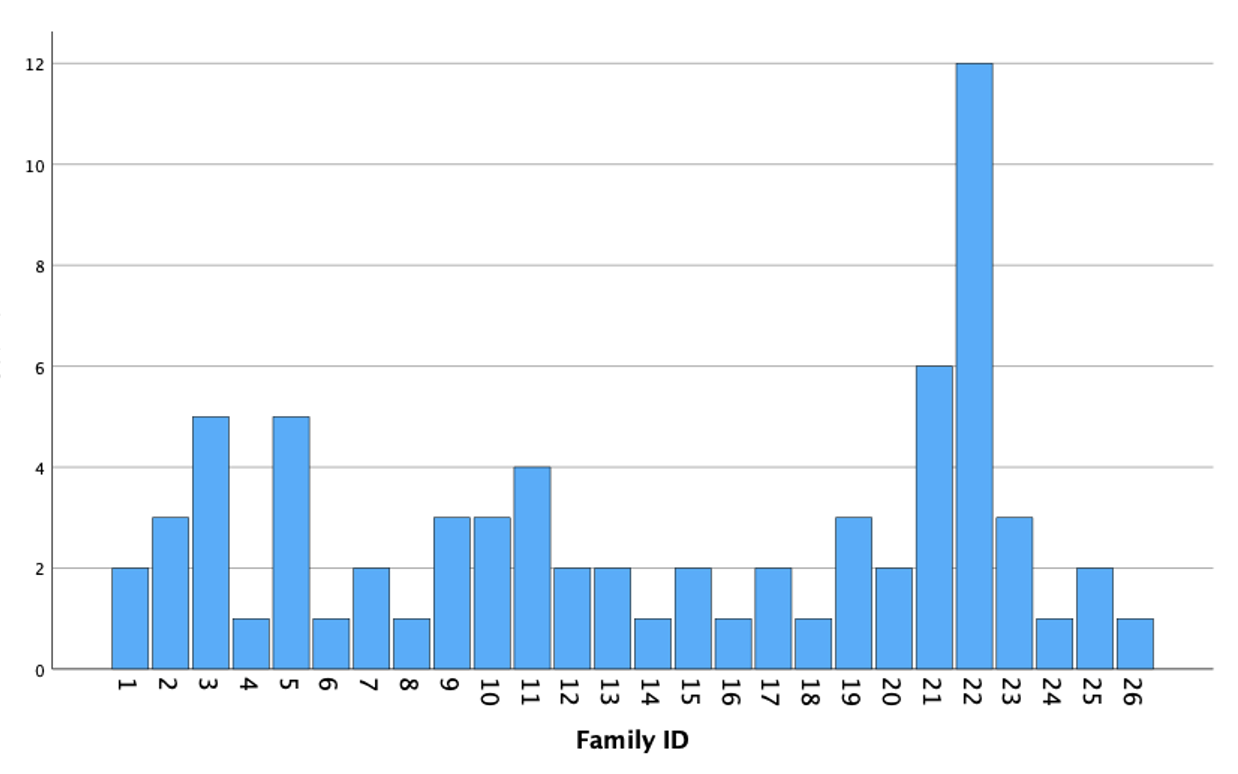


Supplementary Figure 1 Number of FAME study participants in each family.

* One participant belongs to families 21 and 22 and is depicted twice in this graph.


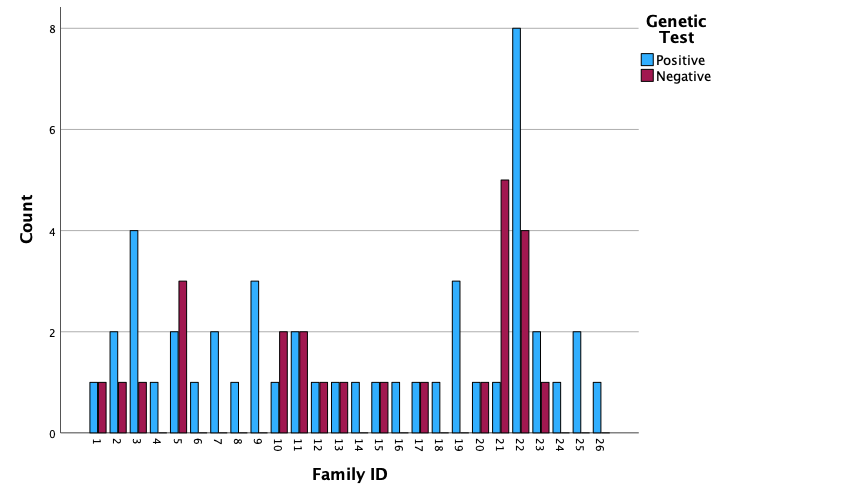


Supplementary figure 2 Genetic test result distribution in each family

Supplementary table1 Distribution of *ALPL* variant in families involved in the FAME study

| Variant | Variant classification | Number of Families | Number of participants tested / Positive for *ALPL* variant |
| --- | --- | --- | --- |
| c.1161A>G p.(Gly387=) | Pathogenic | 5 | 10/8 |
| c.422C>T p.(Thr141Ile) | Likely pathogenic | 1 | 12/8 |
| c.575T>C p.(Met192Thr) | Likely pathogenic | 2 | 6/3 |
| c.571G>A p.(Glu191Lys) | Likely pathogenic | 3 | 5/4 |
| c.881A>C p.(Asp294Ala) | Pathogenic | 1 | 2/2 |
| c.1044_1055del p.(Leu349_Ala352del) | Pathogenic | 1 | 2/1 |
| c.1172G>A p.(Arg391His) | Pathogenic | 1 | 5/4 |
| c.1250A>G p.(Asn417Ser) | Pathogenic | 1 | 1/1 |
| c.1553_1568del p.(Tyr518CysfsTer83) | Likely pathogenic | 1 | 3/2 |
| c.335_340dup p.(Gly112_Thr113dup) | Pathogenic | 1 | 1/1 |
| c.346G>A p.(Ala116Thr) | Pathogenic | 1 | 3/3 |
| c.400_401delinsCA p.(Thr134His) | Pathogenic | 3 | 5/3 |
| c.550C>T p.(Arg184Trp) | Pathogenic | 1 | 1/1 |
| c.295A>G p.(Lys99Glu) | Likely pathogenic | 1 | 3/1 |
| c.215T>C p.(Ile72Thr) | Pathogenic | 1 | 4/2 |
| c.1426G>A p.(Glu476Lys) | Pathogenic | 1 | 2/1 |
| c.920C>T p.(Pro307Leu) | Pathogenic | 1 | 5/1 |
|  |  | 26 | 70/46 |

Supplementary table 2 Comparison of WOMAC, SF-36 questionnaires and HIPS between relatives with positive and negative genetic test

| Womac (n=44) | Positive | Negative |  |
| --- | --- | --- | --- |
| Pain score | 4.5(0,13.75) | 6(1.25,8) | 0.793 |
| Stiffness score | 3.70 (3.06) | 2.42 (2.02) | 0.127 |
| Physical function | 25.5(1.25,46) | 13.5(0.5,30.5) | 0.185 |
| Total score | 39(3,65.75) | 19.5(3.75,43.5) | 0.247 |
| SF-36 (n=44) |  |  |  |
| Physical Functioning | 60(20,98.75) | 72.5(51.25,85) | 0.426 |
| Role-Physical | 37.5(0,100) | 62.5(0,100) | 0.555 |
| Bodily Pain | 46(31,84) | 61.5(41,81.5) | 0.348 |
| General Health | 48.45(23.62531) | 53.4167(30.04622) | 0.552 |
| Vitality | 34.25(21.78091) | 42.5(27.46539) | 0.283 |
| Social Functioning | 56.25(37.5,100) | 62.5(37.5,100) | 0.886 |
| Role Emotional | 33.3333(0,100) | 83.3333(8.3333,100) | 0.344 |
| Mental Health | 60(52,76) | 68(46,88) | 0.266 |
| Physical Health score | 38.9506(15.53534) | 41.5247(12.32867) | 0.543 |
| Mental Health score | 40.9758(13.71963) | 44.6559(12.85726) | 0.364 |
| HIPS (n=44) |  |  |  |
| Have you had a fracture (yes) | 13 (65%) | 11 (45.8%) | 0.2 |
| How many fractures? (n) | 1.15 (1.2) | 0.75 (0.9) | 0.23 |
| Pseudo fracture (n) | 1 (5%) | 1 (4.2%) | 1 |
| Non-vertebral fracture (n) | 3 (15%) | 3 (12.5%) | 0.575 |
| Fractures won't heal (n) | 0 (0%) | 0 (0%) |  |
| Bone pain (n) | 10 (50%) | 6(25%) | 0.086 |
| Hypermobility(n) | 3 (15%) | 1 (4.2%) | 0.237 |
| Joint swelling (n) | 5 (25%) | 3 (12.5%) | 0.249 |
| Joint pain (n) | 10 (50%) | 10 (41.7%) | 0.58 |
| Limiting joint pain (n) | 12 (60%) | 9 (37.5%) | 0.137 |
| Muscle weakness (n) | 7 (35%) | 3 (12.5%) | 0.147 |
| Muscle pain (n) | 9 (45%) | 5 (20.8%) | 0.08 |
| Premature child tooth loss | 5 (25%) | 3 (12.5%) | 0.436 |
| Tooth abscess (n) | 6 (30%) | 3 (12.5%) | 0.261 |
| Excessive cavities(n) | 8 (40%) | 4 (16.7%) | 0.084 |
| Loss of adult teeth (n) | 7 (35%) | 7 (29.2%) | 0.679 |
| Difficult gaining weight (n) | 2 (10%) | 1 (4.2%) | 0.583 |
| Delayed walking (n) | 0 (0%) | 0 (0%) |  |
| Delayed talking (n) | 0 (0%) | 0 (0%) |  |
| Difficult eating/swallowing(n) | 4 (20%) | 0 (0%) | 0.036 |
| Difficult breathing (n) | 0 (0%) | 0 (0%) |  |
| High calcium levels (n) | 0 (0%) | 1 (4.2%) | 1 |
| High phosphate levels(n) | 0 (0%) | 0 (0%) | 0 (0%) |
| Gout (n) | 1 (5%) | 0 (0%) | 0.455 |
| Nephrocalcinosis (n) | 0 (0%) | 1 (4.2%) | 1 |
| Kidney stones (n) | 1 (5%) | 3 (12.5%) | 0.614 |
| Abnormally shaped chest (n) | 0 (0%) | 1 (4.2%) | 1 |
| Abnormal gait (n) | 0 (0%) | 1 (4.2%) | 0.545 |
| Knock knees (n) | 1 (5%) | 1 (4.2%) | 0.708 |
| Bowing legs (n) | 0 (0%) | 0 (0%) |  |
| Short stature (n) | 0 (0%) | 0 (0%) |  |
| Seizure (n) | 0 (0%) | 0 (0%) |  |
| Orthopaedic procedures (n) | 1 (5%) | 1 (4.2%) | 1 |
| Dental procedures (n) | 3 (15%) | 2 (8.3%) | 0.646 |
| Physio/massage/acupuncture (n) | 2 (10%) | 1 (4.2%) | 0.583 |
| Painkillers use (n) | 10 (50%) | 9 (37.5%) | 0.4 |
| Home modified due to HPP (n) | 3 (15%) | 0 (0%) | 0.086 |
| Paid assistance (n) | 1 (5%) | 0 (0%) | 0.455 |
| Use of mobility aids (n) | 6 (30%) | 4 (16.7%) | 0.472 |

Western Ontario and McMaster Universities Arthritis-WOMAC; Short Form of 36 SF-Survey-SF-36; Modified Hypophosphatasia Impact Patient Survey-HIPS; variables are reported as mean (standard deviation) or median (interquartile range).
