# Supplementary material for: Family mapping of previously identified patients with pathogenic or likely pathogenic ALPL variants using predictive genotyping and detailed phenotyping approach: the FAME case-control study
Source: JBMR Plus. 2025 Feb 27;9(5):ziaf034. doi: 10.1093/jbmrpl/ziaf034 (PMC11993272; doi:10.1093/jbmrpl/ziaf034)
Supplement: FAME_study-Supplementary_material_2_ziaf034 [file fame_study-supplementary_material_2_ziaf034.pdf]

FAME study (STH 21685) Modified Hypophosphatasia Impact Patient Survey (HIPS)

This form asks questions about your medical history, mobility and any respiratory issues with particular reference to hypophosphatasia.
v1.0 30Jun21

1. Your initials

____

2. Study ID

____

3. Date

____

Example: 7 January 2019

4. Do other members of your family have hypophosphatasia?

Mark only one oval.

- ( ) Yes
- ( ) No
- ( ) Maybe

5. If yes, how many of your relatives have been diagnosed with hipophosphatasia (HPP)?

____

6. At what age did you first experience symptoms of hypophosphatasia (HPP)?

____

7. What were the first symptoms of hypophosphatasia (HPP) that you experienced? Please gi details

____
____
____
____
____

8. Have you ever been diagnosed with or treated for any of the following conditions? (Please tick all that apply)

Tick all that apply.

☐ Difficulty gaining weight (feeding difficulties as an infant/child)
☐ Delayed walking (first walked at 15 months of age or later)
☐ Delayed talking (speech difficulties)
☐ Short stature (women <5 feet tall, men <5 feet 4 inches)
☐ Seizures
☐ Abnormally shaped chest (rib cage abnormalities)
☐ Abnormally shaped head (skull)
☐ Bowing of legs (rickets in legs)
☐ Bowing of arms (rickets in arms)
☐ Knock knees (knees touch but ankles do not touch when standing upright)
☐ Vertebral fracture (broken bone in back)
☐ Non-vertebral fracture (broken bone anywhere other than the back)
☐ Club foot deformityBone pain (arms, ribs, back, legs, feet) severe enough to force you to limit your activities
☐ Bone pain (arms, ribs, back, legs, feet) severe enough to require pain medication
☐ Fractures that won’t heal
☐ Pseudofractures (incomplete fractures)
☐ Unusual gait or way of walking/running
☐ Extremely flexible joints (hypermobility)
☐ Joint swelling
☐ Joint pain (neck, shoulder, elbow, wrist, hips, knees, ankles)

9. If you experience join pain, is it sever enough to limit your activities?

Mark only one oval.

- Yes
- No

10. If you experience join pain, is it severe enough to require medication?

Mark only one oval.

- Yes
- No

11. Have you ever been diagnosed with or treated for any of the following conditions? (Pleas tick all that apply)

Tick all that apply.

- [ ] Difficulty breathing
- [ ] Pneumonia
- [ ] Premature tooth loss (lost first baby tooth before 5 years of age)
- [ ] Tooth abscess
- [ ] Excessive cavities
- [ ] Loss of adult teeth
- [ ] Difficulty eating/swallowing
- [ ] Muscle weakness
- [ ] Muscle pain
- [ ] Kidney stones
- [ ] Nephrocalcinosis (calcium deposits in the kidneys)
- [ ] High calcium levels in blood
- [ ] High phosphate levels in blood
- [ ] Gout

12. Have you ever broken a bone?

Mark only one oval.

- Yes
- No

If yes, please answer the following questions:

13. When did your first fracture occur?

Mark only one oval.

- Childhood
- Adolescence
- Adult age

14. Approximately how many fractures have you had?

____

15. Please indicate the location of each fracture (foot, arm, leg, back, ribs) and the approxima date of each fracture

____

____

____

____

____

16. How many of the fractures were caused by trauma (impact, force, accident)?

____

17. How many fractures were caused by other reason?

____

18. How many fractures were complete (impact, force, accident)?

____

19. How many fractures were incomplete (pseudo fracture)?

____

20. If you have had incomplete or pseudofractures...How long did it take for the pseudofractures to be diagnosed?

Mark only one oval.

- ( ) Immediately
- ( ) 2 to 4 weeks
- ( ) 1 to 2 months
- ( ) 2 to 3 months
- ( ) Longer than 3 months

21. Approximately how long did it take for the pseudofractures to heal?

Mark only one oval.

- ( ) 1 to 2 months
- ( ) 2 to 3 months
- ( ) 3 to 6 months
- ( ) 6 to 12 months
- ( ) More than 1 year

22. Have you ever had any of the following medical procedures?

Tick all that apply.

- [ ] EMG (test that measures electrical activity in muscles)
- [ ] EEG (test that measures electrical activity in your brain using electrodes)
- [ ] Nerve conduction study (test that measures electrical activity in nerves)
- [ ] Kidney ultrasound
- [ ] Barium swallow study (x-ray of digestive tract after drinking liquid that show up on x-ray)
- [ ] Bone biopsy

23. Have you ever had any of the following surgeries?

Tick all that apply.

- [ ] Skull surgery
- [ ] Stapling of growth plates
- [ ] Fracture fixation with plates and screws
- [ ] Fracture fixation with steel or titanium rods within the bone
- [ ] External fixator
- [ ] Joint replacement (specify joint)
- [ ] Rib replacement
- [ ] Osteotomy (surgical correction of a bone deformity that required cutting into bone)
- [ ] Club foot corrective surgery
- [ ] Dental implant surgery (to replace missing teeth)
- [ ] Root canal surgery

24. If you have had surgery for any complication of hypophosphatasia, please indicate below the reason for the surgery and the approximate date of the surgery

____

____

____

____

____

25. Are you currently receiving any of the following out-patient services?

Tick all that apply.

- [ ] Physical therapy/rehabilitation
- [ ] Respiratory therapy
- [ ] Home health care
- [ ] Massage therapy
- [ ] Acupuncture
- [ ] Occupational therapy
- [ ] Dietary therapy/Nutritional consultation

Medications

26. Please list any PAIN medications you are currently taking including name, dosage and frequency

27. Please list any OTHER medications you are currently taking including name, dosage and frequency

Mobility

28. Has your home been modified due to your hypophosphatasia?

Mark only one oval.

- ⬭ Yes
- ⬭ No

29. If yes, please tick all the areas of your home that have been modified?

Tick all that apply.

- [ ] Kitchen
- [ ] Bedroom
- [ ] Thresholds/Entryways
- [ ] Bathroom

30. Please indicate if you are using paid assistance for the following activities due to hypophosphatasia?

Tick all that apply.

- [ ] Household activities (cleaning, shopping, cooking etc)
- [ ] Family care (watching over children, making appointments etc)
- [ ] Nursing care (medications, equipment etc)
- [ ] Bodily care (bathing, grooming etc)

31. Please indicate which of the following aids you are using or have ever used? (Choose all tl apply)

Tick all that apply.

- [ ] Mechanical lift (car)
- [ ] Handicap ramps
- [ ] Handrails
- [ ] Cane
- [ ] Crutches
- [ ] Adjustable chair (not a wheelchair)
- [ ] Adjustable bed
- [ ] Orthotics (braces)
- [ ] Stander
- [ ] Motorised scooter
- [ ] Walker
- [ ] Manual wheelchair
- [ ] Shower chair
- [ ] Toilet lift
- [ ] Power wheelchair
- [ ] None of the above

32. If you are using a wheelchair, when did you start using your wheelchair (month/ year)?

____

33. If you are using a walking device (cane walker, etc), when did you start using your wheelchair (month/ year)?

____

Respiratory

34. Do you currently use a respiratory support device?

Mark only one oval.

- ◯ Yes
- ◯ No

35. If yes, please tick all that apply

Tick all that apply.

- ☐ Ventilator (with trachea tube)
- ☐ CPAP
- ☐ BiPAP
- ☐ Supplemental oxygen

Other: ☐ ____________________

36. How has your hypophosphatasia developed over the past 5 years?

Mark only one oval.

- ◯ Improved
- ◯ Worsened
- ◯ No change

37. List the 3 symptoms or complications from hypophosphatasia that interfere most with yo[illegible] life

____

____

____

____

____

Thank you for completing this questionnaire

This content is neither created nor endorsed by Google.

Google Forms
